# Comparison of chemoradiotherapy with radiotherapy alone for “biopsy only” anaplastic astrocytoma

**DOI:** 10.18632/oncotarget.17441

**Published:** 2017-04-26

**Authors:** Jing Wu, Ting Zou, Harrison Xiao Bai, Xuejun Li, Zishu Zhang, Bo Xiao, MacLean Nasrallah, Giorgos Karakousis, Ya Cao, Paul J. Zhang, Li Yang

**Affiliations:** ^1^ Department of Neurology, The Second Xiangya Hospital, Central South University, Changsha, Hunan, China; ^2^ Department of Radiology, Hospital of the University of Pennsylvania, Philadelphia, Pennsylvania, United States of America; ^3^ Department of Neurology, Xiangya Hospital, Central South University, Changsha, Hunan, China; ^4^ Department of Pathology and Laboratory Medicine, Hospital of the University of Pennsylvania, Philadelphia, Pennsylvania, United States of America; ^5^ Department of Neurosurgery, Xiangya Hospital, Central South University, Changsha, Hunan, China; ^6^ Department of Radiology, The Second Xiangya Hospital, Central South University, Changsha, Hunan, China; ^7^ Department of Surgery, Hospital of the University of Pennsylvania, Philadelphia, Pennsylvania, United States of America; ^8^ Cancer Research Institute, School of Basic Medicine, Central South University, Changsha, Hunan, China

**Keywords:** chemoradiotherapy, anaplastic astrocytoma, National Cancer Database, radiotherapy, survival

## Abstract

**Background:**

It has become increasingly common to incorporate adjuvant chemotherapy with radiotherapy in the treatment of resected anaplastic astrocytoma based on results from recent phase II/III randomized trials. However, whether or not combined chemoradiotherapy is associated with improved survival outcome in patients who undergo “biopsy only” is less clear.

**Methods:**

The US National Cancer Database was used to identify patients with histologically confirmed, biopsy-only anaplastic astrocytoma who received either radiotherapy alone or combined chemoradiotherapy from 2006 through 2014.

**Results:**

In total, 1896 patients with biopsy-only anaplastic astrocytoma were included, among whom 363 (19.1%) received radiotherapy alone and 1533 (80.9%) received combined chemoradiotherapy. The median age at diagnosis was 60 years. Combined chemoradiotherapy was associated with a significant survival benefit when compared with radiotherapy alone on univariable analysis (median, 13.2 *versus* 5.6 months; hazard ratio [HR], 0.57; 95% confidence interval [CI], 0.50-0.65; *p* < 0.001) and on multivariable analysis (HR, 0.62; 95% CI, 0.55-0.71; *p* < 0.001). A significant survival benefit for combined chemoradiotherapy persisted in a propensity score-matched analysis (HR, 0.67; 95% CI, 0.56-0.78; *p*<0.001).

**Conclusions:**

Our results suggest that combined chemoradiotherapy may be associated with significantly improved survival over radiotherapy alone in patients with anaplastic astrocytoma who undergo biopsy only.

## INTRODUCTION

Anaplastic astrocytoma (AA) is a diffusely infiltrating and malignant primary brain tumor with a median age at diagnosis of 41 years [1, 2]. It constitutes 4% of all malignant central nervous system (CNS) tumors and 10% of all gliomas [3]. AA is defined by the World Health Organization (WHO) as grade III anaplastic glioma [4]. In the new 2016 WHO classification of CNS tumors, WHO grade III AAs are further divided into IDH1-mutant, IDH-wild type and NOS categories [4]. The European Association of Neuro Oncology (EANO) recommends maximal safe surgical resection followed by radiotherapy (RT) alone or chemotherapy (temozolomide [TMZ] or procarbazine, lomustine, and vincristine [PCV]) alone for the treatment of newly diagnosed AA [2]. With conventional treatment, median overall survival (OS) and 5-year survival rates are 3 years and 28%, respectively [3, 5]. Age at diagnosis, neurological function, extent of surgery resection and Karnofsky Performance Status (KPS) have been shown to influence prognosis in adult patients [5–8].

Preliminary results from the CATNON clinical trial showed that concurrent chemoradiotherapy (adjuvant TMZ following RT) was associated with improved survival outcome over RT alone in patients with surgically resected AA [9]. In a recent analysis of 4807 AA patients diagnosed from 2004 to 2014 who underwent surgery using data from the National Cancer Database (NCDB), Shin et al. found that those who received CRT had significantly higher 5-year survival rates than those who received other adjuvant treatment types [10]. However, for about a third of the patients, surgical resection was not the appropriate treatment because the disease was multifocal, their overall health status was poor, or tumor location was difficult to access surgically or in close proximity of critical structures that would increase the chance of procedural morbidity. It is unclear if CRT results in improved OS for AA patients who undergo biopsy only when compared to RT alone. Existing studies, which are often based on a mixture of patients who underwent resection or biopsy, show conflicting results regarding the possible benefit of adding chemotherapy to RT [11, 12].

In this study, we used a large national database to define prognostic factors for survival and to explore the survival benefit of CRT over RT alone in biopsy-only AA.

## RESULTS

### Patient and treatment characteristics

**Figure 1:**
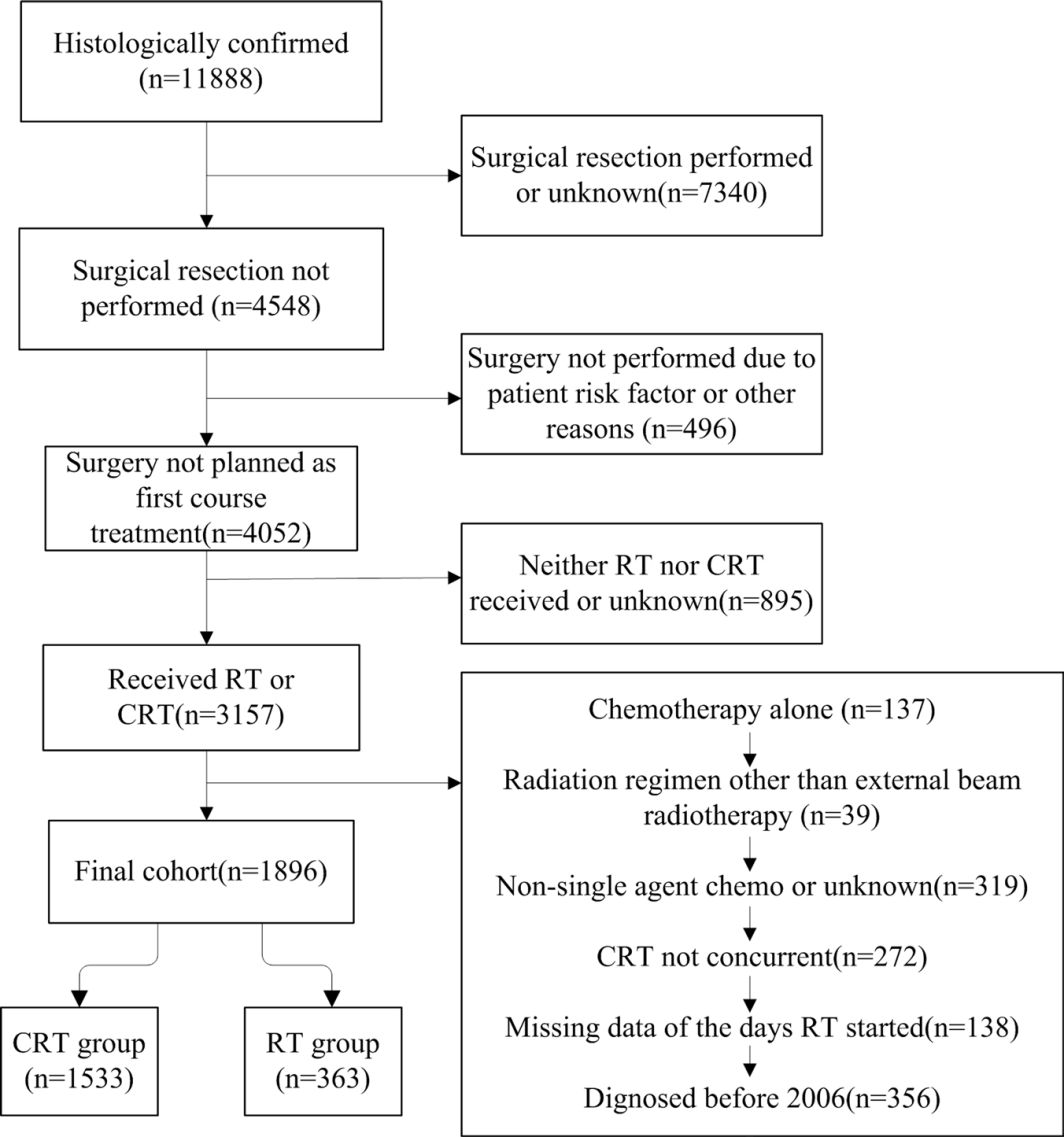
Flow diagram of the current study detailing the patient cohort and the treatment groups

With our initial inclusion criteria, 11888 patients with histologically confirmed AA were identified from the NCDB between 2004 and 2014. Patients whose tumor was surgically resected or whose surgical status was unclear (*n* = 7340) were excluded. Only patients who did not undergo surgical resection due to the reason that surgery was not part of the planned first course treatment (*n* = 4052) were included in the final analysis. Patients were excluded if they were only treated with supportive measures or if they had unknown treatment status (*n* = 895), chemotherapy only (*n* = 137), a nonstandard RT regimen (*n* = 39), multi-agent chemotherapy (*n* = 319), or chemotherapy that took place outside of the 14-day range of RT (*n* = 272). Patients were also excluded if they had missing value of the number of days between the date of diagnosis and the date on which RT was started (*n* = 138), or a diagnosis before 2006 (*n* = 356). In our final cohort, 1896 patients were included, 363 (19.1%) of whom received RT alone, and 1533 of whom (80.9%) received CRT. Our patient selection flowchart is shown in Figure 1.

For the whole cohort, the median age was 60 years (range 3-90); 53.5% of the patients were male, 88.7% were white, and 22.9% had notable co-morbid conditions (Charlson-Deyo score 1 or 2). The median RT starting time, defined as the period between diagnosis was made and RT was initiated, was 28.5 days. The median RT completion period was 44.0 days. The treatment groups did not differ in terms of gender or the median RT starting time. A summary of the characteristics of our cohort is shown in Table 1. On univariable logistic regression analysis, patients were more likely to receive CRT than RT alone if they were younger than 70 years old (*p* < 0.001), white (*p* < 0.001), diagnosed from 2009 to 2014 (*p* < 0.001) or with a Charlson-Deyo score of 0 (*p* = 0.04). Multivariable logistic regression analysis showed that patients were more likely to receive CRT than RT alone if they were younger than 70 years of age (*p* < 0.001), white (*p* < 0.001), or diagnosed from 2009 to 2014 (*p* < 0.001) (Table 2).

**Table 1:** Demographic characteristics of the patient cohort

Characteristics	Number of patients (%)			
	RT (*n*= 363)	CRT (*n*= 1533)	Total (*n*= 1896)	*P*
Age, y				<0.001
Median	66	59	60	
≤49	72(19.8)	464(30.3)	536(28.3)	
50-59	56(15.4)	319(20.8)	375(19.8)	
60-69	80(22.0)	425(27.7)	505(26.6)	
70-79	91(25.1)	251(16.4)	342(18.0)	
≥80	64(17.6)	74(4.8)	138(7.3)	
Gender				0.11
Men	181(49.9)	835(54.5)	1016(53.6)	
Women	182(50.1)	698(45.5)	880(46.4)	
Race				<0.001
White	298(82.1)	1384(90.3)	1682(88.7)	
Black	37(10.2)	87(5.7)	124(6.5)	
other	23(6.3)	52(3.4)	75(4.0)	
unknown	5(1.4)	10(0.7)	15(0.8)	
Diagnosis year				0.001
2006-2008	140(38.6)	456(29.7)	596(31.4)	
2009-2014	223(61.4)	1077(70.3)	1300(68.6)	
Time from diagnosis to start of RT, d				0.87
Median	28	28	28	
≤30	206(56.7)	863(56.3)	1069(56.4)	
>30	157(43.3)	670(43.7)	827(43.6)	
Charlson-Deyo comorbidity score				0.04
0	265(73.0)	1197(78.1)	1462(77.1)	
≥1	98(27.0)	336(21.9)	434(22.9)	
1p/19q co-deletion				0.07
Yes	0(0.0)	17(1.1)	17(0.9)	
No	16(4.4)	70(4.6)	86(4.5)	
Unknown	347(95.6)	1446(94.3)	1793(94.6)	
Tumor size				0.52
≤5cm	161(44.4)	739(48.2)	900(47.5)	
>5cm	51(14.0)	209(13.6)	260(13.7)	
Unknown	151(41.6)	585(38.2)	736(38.8)	
Tumor location				0.20
Frontal lobe	81(22.3)	341(22.2)	422(22.3)	
Temporal lobe	42(11.6)	225(14.7)	267(14.1)	
Parietal lobe	57(15.7)	172(11.2)	229(12.1)	
Occipital lobe	9(2.5)	24(1.6)	33(1.7)	
Brain stem	16(4.4)	48(3.1)	64(3.4)	
Cerebellum	4(1.1)	21(1.4)	25(1.3)	
Ventricle	2(0.6)	6(0.4)	8(0.4)	
Overlapping	57(15.7)	270(17.6)	327(17.2)	
Unknown	95(26.2)	426(27.8)	521(27.5)	
MGMT methylation				1.00
Methylated	6(1.7)	34(2.2)	40(2.1)	
Unmethylated	8(2.2)	47(3.1)	55(2.9)	
Unknown	349(96.1)	1452(94.7)	1801(95.0)	
KPS				1.00
≤70	14(3.8)	45(2.9)	59(3.1)	
>70	10(2.8)	83(5.4)	93(4.9)	
Unknown	339(93.4)	1405(91.7)	1744(92.0)	

Abbreviations: CRT, chemoradiotherapy; RT, radiotherapy; d, day; y, year; KPS, Karnofsky Performance Status.

**Table 2:** Logistic regression to determine odds of receiving chemoradiotherapy versus radiotherapy alone

Variable	Univariable analysis		Multivariable Analysis	
	OR(95% CI)	*P*	OR(95% CI)	*P*
Age: < 70 vs >70 y	2.75(2.15-3.51)	<0.001	2.94(2.29-3.78)	<0.001
Gender: men vs women	0.83(0.66-1.06)	0.11		
Race: white vs nonwhite	2.03(1.48-2.78)	<0.001	2.322(1.67-3.22)	<0.001
Diagnosis year: 2006-2008 vs 2009-2014	0.68(0.53-0.86)	0.001	0.64(0.49-0.81)	<0.001
Time from diagnosis to RT start:>30 vs <30d	1.01(0.81-1.28)	0.87		
Charlson-Deyo score: 0 vs 1-2	1.32(1.01-1.71)	0.04		0.37
Tumor size: >5cm vs <5cm	0.98(0.95-1.01)	0.21		
Location: supratentorial vs infratentorial	1.16(0.69-1.96)	0.56		

Abbreviations: CI, confidence interval; OR, odds ratio; RT, radiotherapy.

### Survival outcome

**Figure 2:**
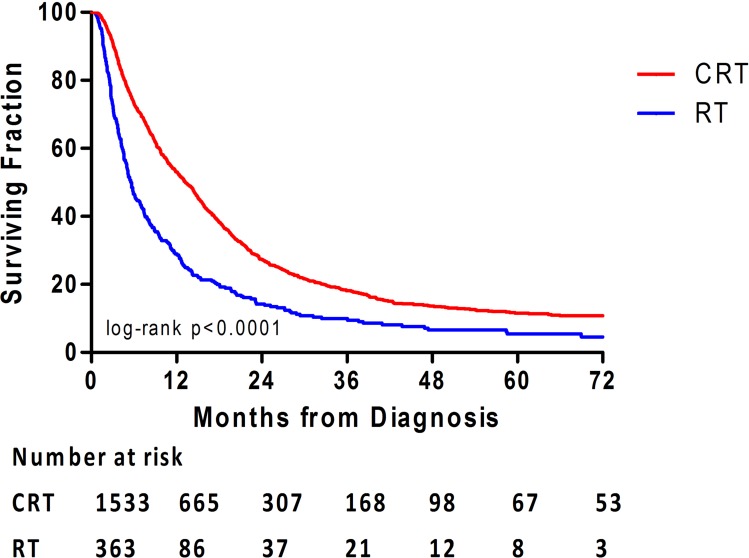
Overall survival is illustrated for patients who received either radiotherapy (RT) alone or chemoradiotherapy (CRT)

The median follow-up time for the entire patient cohort was 10.1 months. Five-year survival rate for the study population was 10.3%. Patients diagnosed at age < 70 years had significantly longer OS than those diagnosed at age > 70 years (median OS: 14.6 *versus* 4.9 months; log-rank *p* < 0.001). Patients receiving CRT had significantly longer OS than patients receiving RT alone (median OS: 13.2 *versus* 5.6 months; log-rank *p* < 0.001). The difference in OS between patients who received CRT and those who received RT alone was statistically significant starting at 6 months and lasted until 36 months after diagnosis. In both treatment groups, long-term survival at 72 months was dismal (4.5% and 10.8%, respectively) (Figure 2, Table 3).

**Table 3:** Overall survival over time by RT and CRT group

Variable	RT alone (*n*= 363)	CRT (*n*= 1533)
Median OS(95% CI),mo	5.6(4.82-6.27)	13.2(12.08-14.33)
OS(%) at		
3mo	72.5(72.45-72.55)	90.8(90.78-90.81)
6mo	46.2(46.14-46.25)	73.2(73.17-73.22)
12mo	28.7(28.65-28.74)	52.9(52.87-52.93)
18mo	19.6(19.55-19.64)	38.2(38.17-38.22)
24mo	14.2(14.16-14.24)	27.4(27.37-27.42)
36mo	9.5(9.46-9.53)	18.2(18.17-18.22)
72 mo	4.5(4.48-4.52)	10.8(10.78-10.81)

Abbreviations: CI, confidence interval; CRT, chemoradiotherapy; OS, overall survival; RT, radiotherapy; mo, month.

After adjusting for differences in age, gender, race, year of diagnosis, Charlson-Deyo score, and median RT starting time, CRT was independently associated with longer OS compared with RT alone (adjusted HR, 0.62; 95% confidence interval [CI], 0.55-0.71; *p* < 0.001). RT beginning > 30 days after diagnosis (adjusted HR, 0.75; 95% CI, 0.67-0.83; *p* < 0.001) was associated with longer OS. Age > 70 years (adjusted HR, 2.48; 95% CI, 2.19-2.82; *p* < 0.01) and a Charlson-Deyo score of 1 or 2 (adjusted HR, 1.43; 95% CI, 1.26-1.61; *p* < 0.001) were associated with shorter OS (Table 4).

**Table 4:** Cox regression demonstrating predictors of overall survival

Variable	Univariable analysis		Multivariable analysis	
	HR(95% CI)	*P*	AHR(95% CI)	*P*
Treatment: CRT vs RT	0.57(0.50-0.65)	<0.001	0.62(0.55-0.71)	<0.001
Age: >70 vs <70 y	2.67(2.36-3.02)	<0.001	2.48(2.19-2.82)	<0.001
Gender: men vs women	1.05(0.94-1.17)	0.37		
Race: white vs nonwhite	1.02(0.86-1.21)	0.83		
Diagnosis year: 2009-2014 vs 2006-2008	0.99(0.88-1.10)	0.81		
Time from diagnosis to RT start:>30 vs <30d	0.75(0.67-0.84)	<0.001	0.75(0.67-0.83)	<0.001
Charlson-Deyo score: 1-2 vs 0	1.61(1.43-1.83)	<0.001	1.43(1.26-1.61)	<0.001
Tumor size: >5cm vs <5cm	0.99(0.97-1.00)	0.11		
Location: supratentorial vs infratentorial	1.00(0.77-1.31)	0.95		

Abbreviations: AHR, adjusted hazard ratio; CI, confidence interval; CRT, chemoradiotherapy; HR, hazard ratio; RT, radiotherapy.

Patients were divided into two subgroups based on age (< 70 *vs*. > 70 years), and subgroup analyses were performed. Consistent with the whole-group analysis, CRT resulted in longer median OS in elderly patients when compared to the RT alone (5.9 *versus* 3.4 months; log-rank *p* < 0.001). In patients older than 70 years, CRT was identified as an independent predictor of longer OS on multivariable analysis (adjusted HR, 0.51; 95% CI, 0.41-0.63; *p* < 0.001). In addition, patients with RT beginning > 30 days (adjusted HR, 0.75; 95% CI, 0.61-0.92; *p* = 0.007) after diagnosis and a Charlson-Deyo score of 0 (adjusted HR, 0.79; 95% CI, 0.64-0.99; *p* = 0.04) were also associated with longer OS (Table 5). Furthermore, in patients aged greater than 70 years with co-morbidity (Charlson-Deyo score of 1 or 2), the difference in OS between patients who received CRT and those who received RT alone remained (HR, 0.59; 95% CI, 0.40-0.88; *p* = 0.01).

**Table 5:** Cox regression to determine predictors of survival in the elderly patients (age>70)

Variable	Univariable Analysis		Multivariable Analysis	
	HR (95% CI)	*P*	AHR (95% CI)	*P*
Treatment: CRT vs RT	0.51(0.41-0.63)	<0.001	0.50(0.40-0.63)	<0.001
Gender: men vs women	1.11(0.90-1.37)	0.31		
Race: white vs nonwhite	0.89(0.60-1.32)	0.57		
Diagnosis year: 2009-2014 vs 2006-2008	0.81(0.65-1.01)	0.06		0.10
Time from diagnosis to RT start:>30 vs <30d	0.74(0.60-0.92)	0.006	0.75(0.61-0.92)	0.007
Charlson-Deyo score: 0 vs 1-2	0.81(0.65-1.00)	0.05	0.79(0.64-0.99)	0.04
Location: supratentorial vs infratentorial	1.11(0.45-2.71)	0.81		

Abbreviations: AHR, adjusted hazard ratio; CI, confidence interval; CRT, chemoradiotherapy; HR, hazard ratio; RT, radiotherapy.

**Figure 3:**
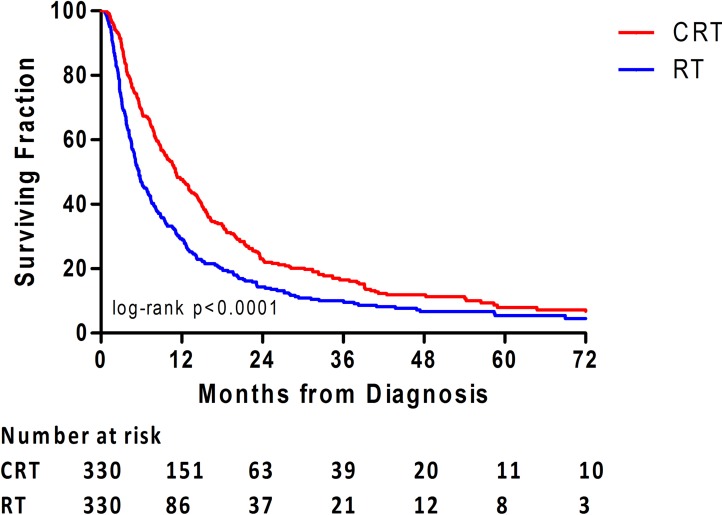
Overall survival is illustrated between the treatment groups for the propensity-matched analysis cohort

Finally, age, race, RT starting time and Charlson-Deyo score were matched between the two treatment groups based on propensity score. Covariates were well balanced as shown in Supplementary Figure 1. In the propensity-matched cohorts, CRT was associated with significantly longer OS than RT alone (median OS: 11.1 *versus* 5.7 months; HR, 0.67; 95% CI, 0.56-0.78; *p* < 0.001) (Figure 3).

## DISCUSSION

CRT or RT alone is regarded as the optimal treatment for AA patients who have undergone surgical resection [2, 13, 14]. However, there is limited data regarding the optimal therapy for patients with AA who only had biopsy. In this study, with a large cohort from a national database, a statistically significant survival benefit was demonstrated in biopsy-only AA patients who were treated with CRT when compared with those who were treated with RT alone.

There is increasing evidence that high-grade gliomas may benefit from CRT [14, 15]. In a meta-analysis of 12 randomized controlled trials comparing RT alone with CRT in high-grade glioma, survival was significantly prolonged with the addition of chemotherapy (HR of 0.85, CI 0.78-0.91, *p* < 0.0001) [2]. However, the randomized controlled trials included in this meta-analysis were mostly based on PCV [2]. Over the past decade, TMZ has become increasingly preferred to PCV because of it is safer profile and tolerability [2, 16]. TMZ damages the DNA by alkylating/methylating DNA at the N-7 or O-6 positions of guanine residues and triggers the death of tumor cells [17]. However, if a tumor cell expresses O6-alkylguanine DNA alkyltransferase encoded in humans by the O-6-methylguanine-DNA methyltransferase (MGMT) gene, it can repair this type of DNA damage, and thus diminishes the therapeutic efficacy of TMZ [17]. Several studies comparing postoperative RT alone and CRT in AA patients have shown conflicting results [11, 12, 18]. First, only a small portion of the patients analyzed in these studies were biopsy-only. Second, the sample size was small and no subgroup analysis on biopsy-only patients was performed. Our study is the first in the literature to compare CRT and RT alone in a large cohort of AA patients who underwent only biopsy.

In our study, CRT was independently associated with longer OS when compared with RT alone. The median OS was 13.2 months for the CRT group, and only 5.6 months for the RT-alone group. The OS was much shorter in our study when compared with the most recent phase II/III randomized trials on anaplastic gliomas because our cohort consisted of 100% biopsy-only patients, while less than 15% of the patients in these trials were biopsy-only [19, 20]. Absolute survival time after diagnosis increased by 7.6 months in the CRT group when compared with the RT along group. This is a clinically significant increase in survival especially considering the poor prognosis for biopsy-only AA patients.

It is a near consensus that increasing age is a negative predictor of survival in patients with gliomas [21]. Previous studies have suggested alternative treatment regimens for elderly patients [22, 23]. In a retrospective study of 42 patients aged greater than 65 years with new-diagnosed AA who underwent surgical resection or biopsy, Tanaka et al. found that CRT was associated with longer OS when compared to RT alone (*p* = 0.01) [24]. On multivariable analysis after adjusting for age, we observed that CRT was still associated with a longer OS when compared with RT alone. Furthermore, in our subgroup analysis of elderly patients, patients receiving CRT had significantly longer OS than those receiving RT alone. However, the absolute benefit of CRT for these patients was only 2.5 months, the clinical significance of which is less clear.

We would like to acknowledge a few limitations of our study. First, there was no central pathology review so presumably some samples may have been misdiagnosed as AA. Second, data on rescue therapies post primary course of treatment was not available in NCDB. Therefore, we could not determine if the two groups were balanced on any therapies (e.g. bevacizumab, reirradiation) received following the primary treatment. Third, the NCDB lacked data on IDH1 mutation and had only limited data on KPS, 1p19q codeletion status and MGMT promoter status which precluded any meaningful analysis. These molecular markers have been shown to be significant prognostic factors in patients with AA [25, 26]. It is difficult to evaluate if the CRT and RT alone treatment groups were balanced on these factors mentioned above. In addition, even though our study of biopsy-only AA patients has demonstrated a survival benefit of CRT over RT alone in a national sample, the inherent selection bias of a retrospective study can only be excluded with a prospective randomized control trial.

In conclusion, our results suggest that CRT may be associated with significantly improved OS over RT alone in patients with AA who undergo biopsy only. Future studies with a randomized design or including subgroup analysis based on molecular data are needed to confirm these results.

## MATERIALS AND METHODS

### Study population

The data used in this study were extracted from NCDB which included hospital registry data set that capture approximately 70% of all newly diagnosed malignancies in the United States. NCDB is a joint project of the Commission on Cancer of the American College of Surgeons and the American Cancer Society. The data used in this study were derived from a deidentified NCDB data file. The American College of Surgeons and the Commission on Cancer have not verified and are not responsible for the analytic or statistical methodology used, or for the conclusions drawn, from these data by the investigators.

Our study population consisted of 1896 patients diagnosed with AA from 2006 to 2014. We only included patients after 2006 since the standard of treatment changed from PCV to TMZ as a consequence of the EORTC-NCIC trial whose results were published in 2005 [16]. The patient population was limited to AA patients who underwent biopsy only without surgical resection, and their diagnosis was confirmed by microscopic histology. Patients were excluded if their diagnosis was not confirmed by histology, or if it was unclear if their tumors were surgically removed. In addition, since single-agent chemotherapy is most consistent with TMZ treatment, we only included patients who received single-agent chemotherapy in the study population. In this study, we defined CRT as combined chemoradiation treatment where chemotherapy was given within 14 days of RT. In the study cohort, radiotherapy refers to external beam radiotherapy only. A summary of our study cohort is shown in Figure 1. We also performed subgroup analyses among the elderly patients (defined as age greater than 70 years) in both cohorts.

### Statistical methods

All data analyses were performed using SPSS 22.0 (SPSS Inc., Chicago, IL). Factors that influenced the liability of receiving either RT alone or CRT were identified by comparing the demographic and clinicopathologic data of patients between RT alone and CRT groups using the chi-square test and the logistic regression multivariate analysis. Patients’ co-morbidity, or the lack of, was evaluated by the Charlson-Deyo score. Charlson-Deyo score (0, 1, or 2) was assigned according to NCDB guidelines based on how many co-morbid conditions were reported and their relative severity.

Kaplan-Meier analyses were used to analyze OS between the RT alone and CRT groups. Univariable Cox regression followed by multivariable Cox proportional hazard regression was used to calculate hazard ratios (HRs) for survival and identify independent prognostic factors for OS. A two-sided p value of < 0.05 was considered statistically significant. To balance confounding factors between groups, we used MatchIt package in R to create matched cohorts based on propensity scores (MatchIt packages in R). Matching was performed using independent factors that had association with survival on univariate cox regression or significantly more likely to be in patients receiving CRT as opposed to RT. Next, the same survival analyses were performed in the matched cohorts.

## SUPPLEMENTARY FIGURE



## Abbreviations

RT: Radiotherapy
CRT: Chemoradiotherapy
OS: Overall Survival
AA: Anaplastic Astrocytoma
