# Supplementary material for: Comparison of chemoradiotherapy with radiotherapy alone for “biopsy only” anaplastic astrocytoma
Source: Oncotarget. 2017 Apr 26;8(40):69038–46. doi: 10.18632/oncotarget.17441 (PMC5620319; doi:10.18632/oncotarget.17441)
Supplement: Supplementary file 1 [file oncotarget-08-69038-s001.pdf]

Comparison of chemoradiotherapy with radiotherapy alone for "biopsy only" anaplastic astrocytoma

SUPPLEMENTARY MATERIALS

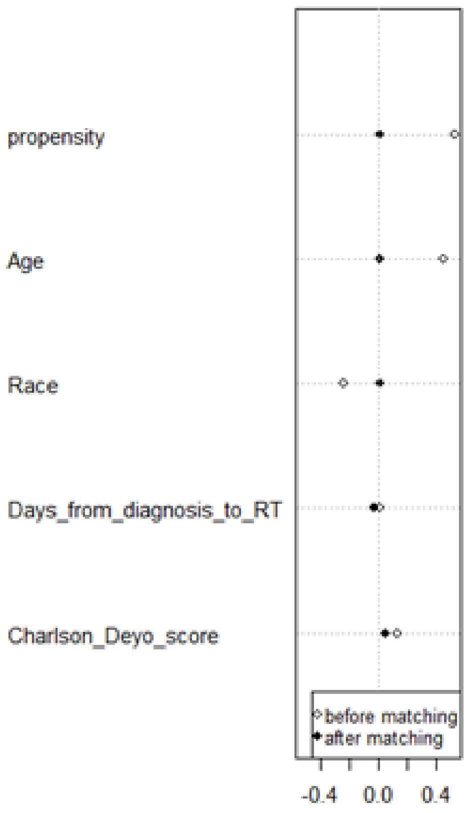

Supplementary Figure 1: Standardized differences of means showing well balanced covariates after propensity score matching.
